# A Detailed Physiologically Based Model to Simulate the Pharmacokinetics and Hormonal Pharmacodynamics of Enalapril on the Circulating Endocrine Renin-Angiotensin-Aldosterone System

**DOI:** 10.3389/fphys.2013.00004

**Published:** 2013-02-08

**Authors:** Karina Claassen, Stefan Willmann, Thomas Eissing, Tobias Preusser, Michael Block

**Affiliations:** ^1^School of Engineering and Science, Jacobs University BremenBremen, Germany; ^2^Computational Systems Biology, Bayer Technology Services GmbHLeverkusen, Germany; ^3^Modelling and Simulation, Fraunhofer MEVISBremen, Germany

**Keywords:** physiologically based pharmacokinetic model, cardiovascular, renin-angiotensin-aldosterone system, enalapril, enalaprilat

## Abstract

The renin-angiotensin-aldosterone system (RAAS) plays a key role in the pathogenesis of cardiovascular disorders including hypertension and is one of the most important targets for drugs. A whole body physiologically based pharmacokinetic (wb PBPK) model integrating this hormone circulation system and its inhibition can be used to explore the influence of drugs that interfere with this system, and thus to improve the understanding of interactions between drugs and the target system. In this study, we describe the development of a mechanistic RAAS model and exemplify drug action by a simulation of enalapril administration. Enalapril and its metabolite enalaprilat are potent inhibitors of the angiotensin-converting-enzyme (ACE). To this end, a coupled dynamic parent-metabolite PBPK model was developed and linked with the RAAS model that consists of seven coupled PBPK models for aldosterone, ACE, angiotensin 1, angiotensin 2, angiotensin 2 receptor type 1, renin, and prorenin. The results indicate that the model represents the interactions in the RAAS in response to the pharmacokinetics (PK) and pharmacodynamics (PD) of enalapril and enalaprilat in an accurate manner. The full set of RAAS-hormone profiles and interactions are consistently described at pre- and post-administration steady state as well as during their dynamic transition and show a good agreement with literature data. The model allows a simultaneous representation of the parent-metabolite conversion to the active form as well as the effect of the drug on the hormone levels, offering a detailed mechanistic insight into the hormone cascade and its inhibition. This model constitutes a first major step to establish a PBPK-PD-model including the PK and the mode of action (MoA) of a drug acting on a dynamic RAAS that can be further used to link to clinical endpoints such as blood pressure.

## Introduction

Hypertension is a worldwide epidemic, which affects all ages and racial populations. It has a very high incidence and is the leading cause of cardiovascular mortality (Roger et al., 2012). There is still a lack of mechanistic models in the published literature that integrate the available physiological knowledge and relate the pharmacokinetics (PK) of hypertension drugs to their pharmacodynamics (PD) effects. One model addressing among others the RAAS and the blood pressure, is the Guyton’s physiological model (Guyton et al., 1972), it was extended and published recently (Montani and Van Vliet, 2009; Osborn et al., 2009). Although this and other models may contain all the parameters listed in this present study and although they might have been validated against several physiological situations they are not based on a generic whole body physiologically based PK (wb PBPK) background model. Other approaches concerning the hormone cascade, as for example by White et al. (1989), Levitt and Schoemaker (2006), Guillaud and Hannaert (2010), Zhou et al. (2012), have different focuses or approaches.

**Figure 1:**
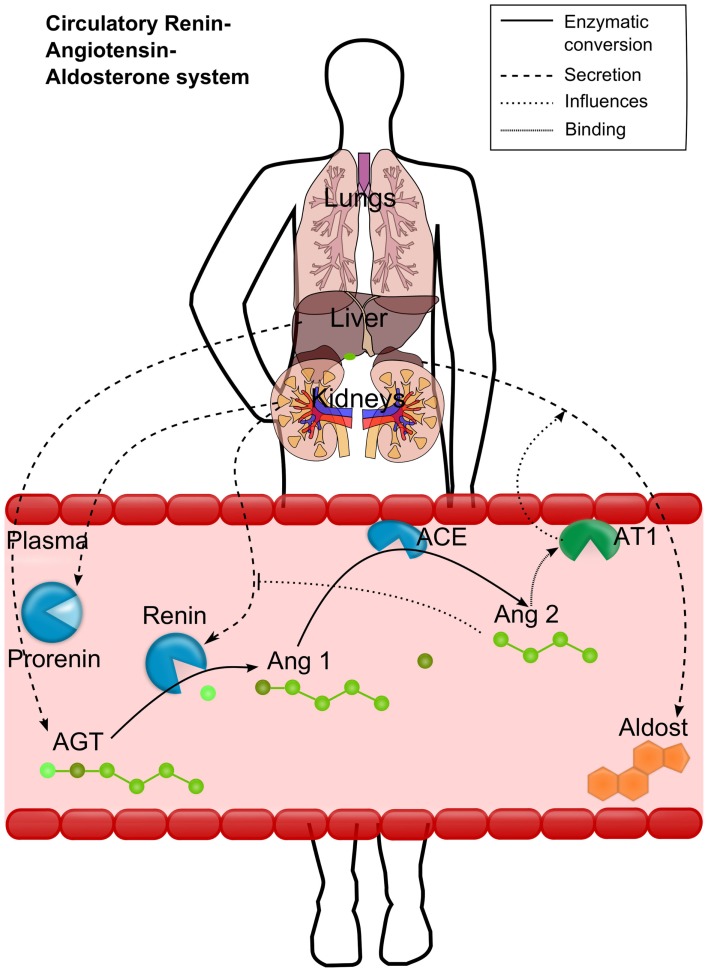
**Schematic representation of the renin-angiotensin-aldosterone system (RAAS)**. Renin converts the hepatically secreted angiotensinogen (AGT) to angiotensin 1 (Ang1). Ang1 is then converted to angiotensin 2 (Ang2) by the membrane bound angiotensin-converting-enzyme (ACE). Ang2 then binds to the angiotensin 2 receptor type 1 (AT1). The most important effects of AT1 binding are depicted either: the aldosterone secretion by the adrenals and the inhibition of renin activation from prorenin. Different processes are depicted by different line styles (see upper right box).

To overcome the lack of detailed physiologically based models for cardiovascular PD, we aimed to establish a mechanistic model for the RAAS covering all relevant biological processes depicted in Figure 1. Renin is synthesized from the enzyme precursor prorenin, which is produced mainly in the kidney and secreted into the plasma by the granular cells (Krop et al., 2008). The active enzyme renin is stored in the cells of the juxtaglomerular apparatus is released immediately on stimulation of these cells (Danser et al., 1998). After secretion into the plasma, renin converts the hepatically synthesized inactive hormone angiotensinogen (AGT) to angiotensin 1 (Ang1). Ang1 is then converted in the plasma compartment by the membrane bound angiotensin-converting-enzyme (ACE), expressed by the vascular endothelium, to angiotensin 2 (Ang2). Ang2 has an inhibiting effect on renin synthesis and secretion and thus on the plasma levels of Ang1 and Ang2 (Johns et al., 1990). Ang2 is a vasoactive peptide that induces volume (and Na) retaining effects as well as vasoconstricting effects and an increase in blood volume, leading to increased blood pressure. It potentiates aldosterone secretion by interacting with its angiotensin 2 receptor type 1 (AT1). Aldosterone increases the blood pressure as well (Gornall et al., 1960) by sodium and extracellular volume retention. Several cardiovascular disease states are associated with changes in circulating Ang2 (Laragh et al., 1972; Laragh, 1995).

Inhibitors of the RAAS can be classified by their mode of action (MoA; Cagnoni et al., 2010; Eisenberg, 2011; Aronson and Krum, 2012) into five categories:

ACE inhibitors (ACEIs),
Direct renin inhibitors (DRIs),
Angiotensin-2-receptor-blockers (ARBs),
Aldosterone synthase inhibitors (ASIs), and
Potassium sparing diuretics.

A common drug of the first type of inhibitors is enalaprilat (Enaat), a potent and reversible ACE inhibitor (MacFadyen et al., 1993; Dhareshwar, 2007). Enaat is poorly absorbed from the gastrointestinal tract. Therefore, an ethyl ester promoiety was incorporated within the administered parent drug enalapril (Ena) to increase its oral bioavailability. After absorption of inactive Ena by the peptide carrier-mediated transport system in the intestine, the prodrug Ena is converted to Enaat by the carboxylesterase family of enzymes activating it for ACE inhibition (MacFadyen et al., 1993; Dhareshwar, 2007).

## Materials and Methods

A model of the RAAS based on available information was established (Eissing et al., 2011). To evaluate the RAAS model, a generic PBPK model of Ena and Enaat was developed and validated with clinical data that was then coupled to the RAAS model.

### Software

The RAAS model was created using the Systems Biology software suite that consists of the wb PBPK software tool PK-Sim^®^ (Version 4.2, Bayer Technology Services, Leverkusen, Germany) and the general purpose modeling software MoBi^®^ (Version 2.3, Bayer Technology Services, Leverkusen, Germany). This software has successfully been applied for diverse modeling studies in laboratory animals and humans (Willmann et al., 2003, 2004, 2007, 2010; Edginton et al., 2006a,b,c, 2008; Brochot et al., 2010; Eissing et al., 2011). The parameter identifications for MoBi models were done using MATLAB^®^ (R2010b, The MathWorks, Inc., Natick, MA, USA) and the MoBi Toolbox for MATLAB. Identification algorithm for Ena and Enaat models was Monte Carlo, while RAAS identification algorithm was fminsearch (fminsearch uses the Nelder–Mead Simplex method). The identification error function was in both cases error root mean square. For additional information, see the software manual or http://www.systems-biology.com/products.

### Development of the RAAS-PBPK model

Physiological data presented in Table 1 contains the information gained from the literature or, if not available by optimization based on data for Ang1, Ang2, aldosterone, renin, prorenin, ACE, AGT, and the AT1. Optimization was done with the overall coupled model and with all RAAS parameters (Table 1) simultaneously. For each of them an individual PBPK model was developed which contains many physiological background parameters (Eissing et al., 2011) that are derived from the few input parameters presented in Table 1. Mass transfer within the plasma (*r*_1_) between organs is calculated as organ specific blood flow (*Q*_organ_) times the drug concentration within the plasma of the respective organ (*C*_pls___organ_) times the overall plasma fraction (1 − HCT) which is one subtracted by the hematocrit (HCT) value. Passive transport into the interstitial and intracellular space is realized by multiplying organ specific partition coefficients *K*_org_.

(1) $$r_{1}=Q_{\text{organ}}\cdot \left(1-\text{H}\;\text{C}\text{T}\right)\cdot C_{\text{pls\_organ}}$$

**Table 1:** **Parameters and variables used for the different actors in the RAAS**.

Parameter/variable name	Value	Unit	Reference
**AGT^†^**			
Molecular weight	53.15	kDa	UniProt Consortium (2012)
Plasma concentration*	0.60	μmol· L^−1^	In agreement with Kobori et al. (2009) and Wang et al. (2012), consistent with the range given by Katsurada et al. (2007)
*t*_1/2_	11.29	min	Optimization
*k*_Secretion_	7.81	L· min^−1^	Optimization
**RENIN^†^**			
Molecular weight	48	kDa	Sealey et al. (1980)
Plasma concentration*	3.62E−07	μmol· L^−1^	Consistent with Juillerat et al. (1990), Nussberger et al. (2002)
*k*_cat_	3.60	min^−1^	Optimization
*K*_m_	5.04E−02	μmol· L^−1^	Optimization
*t*_1/2_	1.44	min	Optimization
**ACE**			
Molecular weight	180	kDa	Thevananther and Brecher (1999)
*k*^+^	35.16	L· μmol^−1^· min^−1^	Results from IV Ena model
*k*^−^	2.15E−02	min^−1^	Results from IV Ena model
*k*_cat_	4.48E−04	min^−1^	Optimization
*K*_m_	4.12E−03	μmol· L^−1^	Optimization
**Ang1^†^**			
Molecular weight	1.30	kDa	Wang et al. (2012)
Plasma concentration*	7.91E−06	μmol· L^−1^	Consistent with Juillerat et al. (1990), Nussberger et al. (2002)
*t*_1/2_	0.72	min	Optimization
**Ang2^†^**			
Molecular weight	1.05	kDa	Noda et al. (2006)
Plasma concentration*	4.84E−06	μmol· L^−1^	Consistent with Juillerat et al. (1990), Nussberger et al. (2002)
Log *P*	−1.7	–	Wang et al. (2012)
*K*_inhibition_	1.87E−08	μmol· L^−1^	Optimization
*t*_1/2_	1.54	min	Consistent with Boyd et al., 1967)
**AT1**			
Molecular weight	41	kDa	UniProt Consortium (2012)
*C*_Ref_	7.69	μmol· L^−1^	Optimization
*k*^+^	23.50	L· μmol^−1^· min^−1^	Optimization
*k*^−^	3.07E−02	min^−1^	Optimization
**ALDOST^†^**			
Molecular weight	0.36	kDa	Wang et al. (2012)
Plasma concentration*	2.07E−04	μmol· L^−1^	Consistent with Juillerat et al. (1990), Nussberger et al. (2002)
Log *P*	1.08	–	Wishart et al. (2009)
Protein binding	50	%	Pardridge (2011)
*k*_Production_	1.51E−06	μmol· min^−1^	Optimization
*k*_Secretion_	7.81	L· min^−1^	Optimization
*t*_1/2_	12.87	min	Optimization
**PRORENIN^†^**			
Molecular weight	57	kDa	Sealey et al. (1980)
Plasma concentration*	1.21E−06	μmol· L^−1^	Based on Tu et al. (2012) with a concentration of 69 pg· ml^−1^
Renal plasma concentration intracellular	1.1E−04	μmol· L^−1^	Optimization
*V*_MaxKid_	1.62	μmol· min^−1^· L^−1^	Optimization
*K*_mKid_	4.68E−02	μmol· L^−1^	Optimization
*k*_Secretion_	4.68E−02	L· min^−1^	Optimization
*V*_Kidney_ (intracellular volume kidney)	0.24	L	PK-Sim^®^
*t*_1/2_	5.07	min	Optimization
**CARBOXYLESTERASE**			
*K*_mLiv_	710	μmol· L^−1^	Tabata et al. (1990), Abu-Zahra and Pang (2000)

*Plasma concentration in venous blood at pre-administration steady state.

*^†^Are assumed to circulate only in the plasma*.

The interactions in the coupled model were described as depicted in Figure 1 reflecting the biological processes as detailed in the following. AGT, prorenin, renin, and aldosterone are the four hormones and enzymes synthesized in cells and then secreted endocrinally. It was assumed that these hormones and enzymes are produced intracellular and secreted into the plasma by a first order rate (*r*_2_ in μmol· min^−1^) proportional to the intracellular concentration (*C*_CellularHormone_) in the respective organ with the rate constant *k*_Secretion_ (in L· min^−1^). The renin secretion *r*_3_ (μmol· min^−1^) is described by a “competitive” inhibition equation including the conversion of prorenin to renin influenced by Ang2 (see Figure 1), describing the conversion from prorenin to renin, and simultaneously describing the secretion from intracellular to plasma compartment of the kidney:

(2) $$r_{3}=\frac{V_{\text{MaxKid}}\cdot V_{\text{Kidney}}\cdot C_{\text{Prorenin}}\left(t\right)}{C_{\text{Prorenin}}\left(t\right)+K_{\text{mKid}}\cdot \left(1+\left(\frac{C_{\text{Ang2}\left(t\right)}}{K_{\text{inhibition}}}\right)\right)}$$

*K*_mKid_ and *V*_MaxKid_ are the respective enzymatic activity constants, *V*_kidney_ is the intracellular volume of the kidney, *C*_Prorenin_ is the renal intracellular prorenin concentration (parameters under prorenin in Table 1). *K*_inhibition_ represents the inhibitory constant for Ang2 and *C*_Ang2_ is the renal plasma concentration of Ang2. The degradation rate *r*_4_ (μmol· min^−1^· L^−1^) is described by Guillaud and Hannaert (2010):

(3) $$r_{4}=C\left(t\right)\cdot \left(\frac{\ln2}{t_{1/2}}\right)$$

*t*_1/2_ determines the half-life and *C*(*t*) being the respective plasma concentration over time. These half-lives were optimized for the hormones and enzymes (see Table 1). The conversion of AGT to Ang1 and from Ang1 to Ang2 take place in the plasma compartments of all organs (Eissing et al., 2011) and is an enzymatically regulated process described by a Michaelis–Menten equation (Gould et al., 1980; Ehlers and Kirsch, 1988). The change of hormone mass over time (*r*_5_ in μmol· min^−1^) then depends on the enzyme concentration (*C*_0_) and the prohormone (*C*_Prohormone_) concentration in the respective organs, as well as on the enzyme activity represented by the *K*_m_ and *k*_cat_ of the enzyme (Meyer et al., 2012). This can be expressed by:

(4) $$r_{5}=\frac{k_{\text{cat}}\cdot V_{0}\cdot C_{0}\left(t\right)\cdot C_{\text{Prohormone}}\left(t\right)}{C_{\text{Prohormone}}\left(t\right)+K_{\text{m}}}$$

The calculation refers to the respective organ plasma volumes *V*_0_ in L (taken from PK-Sim^®^ database). ACE is most notably bound to the endothelium (Dzau et al., 2001) and thus not circulating in the model. The concentration of ACE and AT1 in each organ (*C*_0_) is calculated via a reference concentration (*C*_Ref_, see Table 3) times the percentage of reference expression (*E*_Ref_) from literature (see Table 4, factor *E*_Ref_ = 100 for the highest expression value):

(5) $$C_{0}=C_{\text{Ref}}\cdot \frac{E_{\text{Ref}}}{100}$$

Ang2 binds to the AT1 receptor, which influences the aldosterone secretion. AT1 binding rate (*r*_7_ in μmol· min^−1^· L^−1^) is described by a typical equation with *k*^+^ and *k*^−^ binding constants for association and dissociation and the respective concentrations for AT1 (*C*_0_), Ang2 (*C*_2_), and the AT1-Ang2 complex (*C*_Complex_):

(6) $$r_{7}=\left(k_{+}\cdot C_{0}\cdot \left(t\right)\cdot C_{2}\left(t\right)\right)-\left(k\text{\_}\cdot C_{\text{complex}}\left(t\right)\right)$$

The AT1 concentrations were calculated as explained in Eq. 6, the respective relative expression values (*E*_Ref_) are shown in Table 4, and the reference concentration (*C*_Ref_) is presented in Table 1. As shown in Figure 1 AT1 is membrane bound too and thereby does not circulate in the plasma. Aldosterone synthesis (*r*_8_ in μmol· min^−1^) in our model was directly linked to the complex formation of Ang2 bound to the AT1 (*C*_Complex_), which is modeled as

(7) $$r_{8}=\left(k_{\text{secretion}}\cdot C_{\text{complex}}\left(t\right)\right)+k_{\text{production}}$$

The secretion is thereby described to be dependent on intracellular concentration of Ang2 receptor complex multiplied by the rate constant *k*_Secretion_ (in L· min^−1^) and additionally represents the continuous secretion as *k*_Production_ (Veldhuis et al., 2008).

### Development of the enalapril/enalaprilat PBPK model

A PBPK model for oral Ena including the metabolism to active Enaat was developed first and used as test case for the dynamic behavior of the RAAS model. Based on intravenous (IV) data, parameters for Enaat were determined and then used in the parent-metabolite model. The required physicochemical input properties and clearance processes for the model are presented in Table 2. The model simulations for oral Ena were compared to different experimental data (Biollaz et al., 1982; Noormohamed et al., 1990; Nussberger et al., 2002; Lee et al., 2003; Najib et al., 2003; Gu et al., 2004; Lu et al., 2009). The six different subject and dataset properties (all included to the respective simulations) are shown in Table 3. Only the five parameters presented in Table 3 were optimized with MATLAB^®^. The respective IV Enaat simulations were compared to experimental data of Enaat plasma concentrations after IV Enaat (Hockings et al., 1986) administration. The coupling of parent and metabolite was realized with a hydrolytic reaction in the hepatocytes (Dhareshwar, 2007). This reaction is mediated by different carboxylesterase enzymes. *K*_m_ values for metabolism to Enaat in Table 1 were taken from measurements in rat hepatocytes (Tabata et al., 1990; Abu-Zahra and Pang, 2000). The conversion rate (*r*_9_ in μmol· min^−1^) from Ena into Enaat in the hepatocytes with the respective intracellular volume *V*_Liver_ (see Table 3), the concentrations of Ena (*C*_Ena_) and Enaat (*C*_Enaat_) is modeled by Michaelis–Menten kinetics:

(8) $$r_{9}=\frac{V_{\max\text{Liv}}\cdot V_{\text{Liver}}\cdot C_{\text{Ena}}\left(t\right)}{C_{\text{Ena}}\left(t\right)+K_{\text{mLiv}}}$$

**Table 2:** **Physicochemical and PK properties of enalapril and enalaprilat**.

	Enalapril	Enalaprilat	Unit	Reference
Molecular weight	376.5*	348.4*	g· mol^−1^	Wang et al. (2012)
Log *P*	0.07*	−0.74*	Log units	Ranadive et al. (1992), Remko (2007)
pKa	3.74 (4.75*)	2.03*	–	Remko (2007)
		4.03*	
Plasma protein binding	45 (50*)	50–60 (50*)	%	Sirianni and Pang (1998), Knox et al. (2011)
Hepatic clearance	354	–	ml· min^−1^	Noormohamed et al. (1990)
Renal clearance	18	8–9.5	L· h^−1^	Dhareshwar (2007)

*Values used in the model.

**Table 3:** **Population specific information about subjects, optimized parameters, and model specific parameters**.

	Biollaz et al. (1982)	Gu et al. (2004)	Lee et al. (2003)	Lu et al. (2009)	Najib et al. (2003)	Noormohamed et al. (1990)
No. of individuals	12	20	12	20	24	12
Male/female	12/0	–	–	20/0	24/0	12/0
Age (years)	22–33	–	–	–	23.25 ± 4.55	21–25 (mean 22)
Weight (kg)	–	–	–	–	73.38 ± 9.39	56–80 (mean 70)
Height (cm)	–	–	–	–	175.96 ± 7.66	–
Healthy	Yes	Yes	Yes	Yes	Yes	Yes
Dose (mg)	10	10	20	2·5*	20	20
Fasted	Yes	No	No	Yes	Yes	Yes
**OPTIMIZATION RESULTS**						
*V*_maxLiv_ (μmol· min^−1^· L^−1^)^†^	184.87	178.18	131.88	148.05	146.50	194.36
Renal clearance Ena (L· min^−1^·kg^−1^)	6.02E−03	4.51E−3	6.53E−03	5.02E−03	4.61E−03	5.50E−03
Renal clearance Enaat (L· min^−1^· kg^−1^)	2.99E−04	8.01E−4	4.99E−04	6.30E−04	6.37E−04	6.21E−04
ACE ref conc. (*C*_Ref_) (μmol· L^−1^)	2.59	1.86	4.32	1.30	2.14	4.15
**PARAMETERS OBTAINED FROM PK-Sim^®^ ACCORDING TO DIFFERENT AGE GROUPS**						
*V*_Liver_ (L)	1.56	1.57	1.57	1.35	1.49	1.42

*Two enalapril maleate capsules were administered at the same time, containing 5 mg enalapril each.

*^†^*V*_maxLiv_ values for carboxylesterase enzyme and the conversion from Ena to Enaat in Eq. 8*.

The respective parameters for Michaelis–Menten kinetics are *V*_MaxLiv_ and *K*_mLiv_.

### Assembling of the models to a full dynamic PBPK-RAAS model

To couple the drug model to the full RAAS model the MoA was included. Enaat binds to ACE in the RAAS (see Table 1, ACE parameters) generating an inactive complex. The formation rate of Enaat-ACE complex (*C*_Complex_) is realized as presented in equation 6 for all plasma compartments. ACE concentration is based on the relative expression value in Table 4 which was obtained from Unigene (2010) and processed as described in Meyer et al. (2012). The inhibition of ACE occurs by ACE binding to Enaat, forming an ACE-Enaat- complex, thereby blocking ACE for the interaction with Ang1. The binding is implemented in the plasma compartment of each organ.

**Table 4:** **Reference expression values of ACE per organ as included in PK-Sim 5^®^ expression database (Meyer et al., 2012) from Unigene (2010)**.

Name	ACE	AT1
Gene ID	1636	185
Hs	654434	477887

**Organ**	**Expression *E*_Ref_ in% (absolute value) of highest expression value**	

Brain	15 (12.96)	5 (0.20)
Fat	6 (5.22)	0 (0)
Gonads	2 (2.01)	4 (0.16)
Heart	0 (0.16)	9 (0.35)
Kidney	0 (0.41)	100 (3.85)
Large Intestine	2 (1.44)	27 (1.04)
Liver	0 (0)	76 (2.94)
Lung	100 (86.38)	6 (0.22)
Muscle	0 (0.12)	8 (0.30)
Pancreas	0 (0.23)	0 (0)
Plasma	6 (5.55)	0 (0)
Skin	0 (0)	57 (2.19)
Spleen	0 (0)	89 (3.41)
Small Intestine	1 (0.32)	27 (1.04)
Stomach	3 (2.86)	0 (0)

## Results

### Oral administration of enalapril

**Figure 2:**
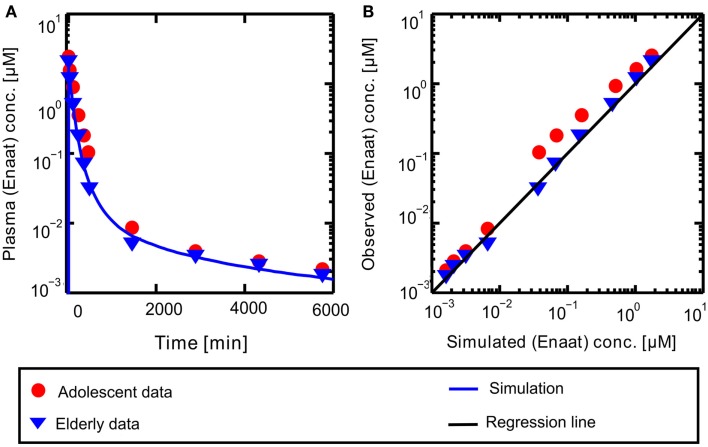
**(A)** Plasma concentration-time profiles of Enaat after IV Enaat administration. The solid blue line is the simulation result compared to the data of Hockings et al. (1986) which are indicated as points (adolescents) and triangles (elderly). **(B)** The simulated versus observed plot for the same simulation with the Hockings data are shown, indicated as points (adolescents) and triangles (elderly). The solid black regression line denotes the line of identity (observed outcome is equal to simulated outcome).

A PBPK model of IV Enaat has been developed with the data indicated in Table 2. In Figure 2 the resulting PK-simulations for IV Enaat are shown and the goodness-of-fit of the model based on data is depicted (Hockings et al., 1986). This model was used to determine the parameters related to Enaat, which are log *P*, Molecular weight, pKa, plasma protein binding and clearance (see Table 2). Additionally the constants of ACE binding were identified (see Table 1). The constant for association is much higher than the dissociation constant, as stated by Reynolds (1984). This parameterization was used within the coupled model without changes.

**Figure 3:**
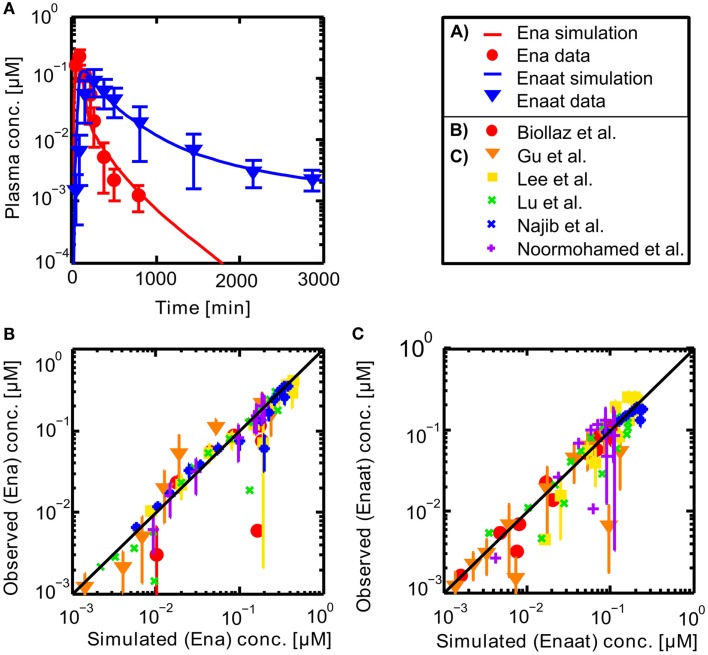
**(A)** Exemplary plasma concentration-time curves of Ena and Enaat from the coupled Ena-Enaat model after oral Ena application compared to experimental data (Gu et al., 2004). The red solid line is the simulated Ena plasma concentration, while the blue solid line is the simulation result of Enaat. The respective data of Ena are indicated as circles, of Enaat as triangles. **(B)** Simulated versus observed plot for Enalapril from the coupled model, compared to the data by Biollaz et al. (1982), Noormohamed et al. (1990), Lee et al. (2003), Najib et al. (2003), Gu et al. (2004), Lu et al. (2009), with SD. The solid black regression line denotes the line of identity (observed outcome is equal to simulated outcome). **(C)** Predicted versus observed plot for Enalaprilat from the coupled Ena-Enaat model, compared to the data by Biollaz et al. (1982), Noormohamed et al. (1990), Lee et al. (2003), Najib et al. (2003), Gu et al. (2004), Lu et al. (2009). The solid black regression line denotes the line of identity (observed outcome is equal to simulated outcome).

Resulting plasma concentrations of ACE based on the explained calculation (peripheral venous plasma concentration in the model validated by data from Gu et al. (2004): 7.17E−4 μmol· L^−1^) correspond nicely with literature data of 2.52E−3 μmol· L^−1^ by Brice et al. (1995). After integrating the metabolite model into the oral model, the coupled model exhibits the behavior presented in Figure 3. Including the population dependent parameters for the different publications (see Table 3), the six model simulations show excellent agreement with the data. The serum concentration profile of Enaat exhibits a prolonged terminal phase, apparently representing the small fraction of the administered dose that has been bound to ACE. As obvious from Figure 3, the model Ena simulations and Enaat predictions accurately fit the observed data.

### RAAS inhibition by enalaprilat

**Figure 4:**
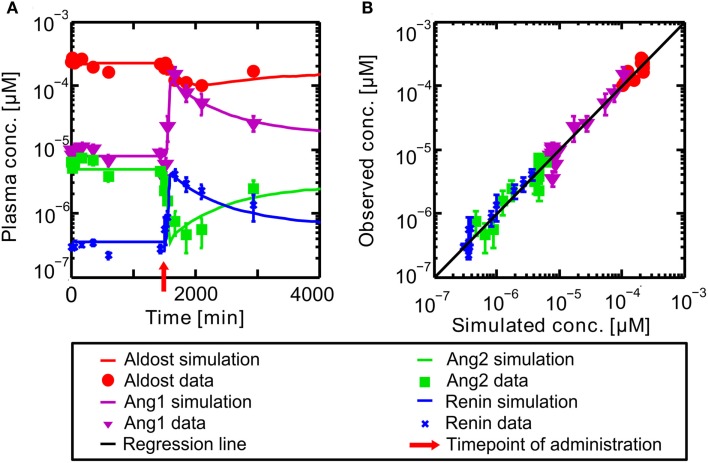
**(A)** Plasma concentration-time curves of hormones included into the RAAS. Hormones are in a steady state until administration of oral Ena at *t* = 1500 min indicated by the red arrow. The respective data are indicated by different symbols (Nussberger et al., 2002). **(B)** Simulated versus observed plasma concentrations of different actors of the RAAS before and after inhibition of ACE by Ena. The different hormones are depicted by different symbols (Nussberger et al., 2002) with their SD. The solid black regression line denotes the line of identity (observed outcome is equal to simulated outcome).

The PBPK model for the circulating, endocrine RAAS was developed to describe the steady state described in literature. It corresponds to the pre-administration steady state discussed further in the following. The model for Ena was then coupled to the RAAS model to explore the dynamical behavior of the system under inhibition. In Figure 4 the results for the full RAAS model under administration of 10 mg Ena are shown at *t* = 1500 min (administration at *t* = 1500 indicated by the red arrow). All parameters of the Ena-Enaat model were used without changes. The pre- and post-administration steady states as well as the dynamical transition after administration at *t* = 1500 min (red arrow) show excellent agreement to literature data (Table 1, Figure 4A). In detail, Figure 4 shows the MoA of Ena. After the administration of Ena, ACE is inhibited. As can be seen, the conversion of Ang1 to Ang2 is inhibited, lowering the Ang2 and aldosterone plasma levels. The negative feedback loop from the Ang2 to the renin synthesis is inhibited, leading to an increase of the renin plasma concentration, and thereby the Ang1 levels. The comparison of these induced changes in plasma levels by a scatter plot (Figure 4B) to study data from Nussberger et al. (2002) shows good agreement and corresponds also to older experimental data (Given et al., 1984; Johnston et al., 1984; Juillerat et al., 1990; Azizi et al., 1997).

The simulation of exemplary Ena administration shows, that the full dynamical behavior of inhibition and the other RAAS-hormone levels can be represented accurately (see Figure 4).

## Discussion

A full mechanistic model of the circulating RAAS was developed. The model represents the steady state and dynamic behavior of the RAAS in an accurate manner. This was demonstrated by simulating the effects of representative drug acting on the system. The Ang1 concentration is influenced by different reactions, Ang1 generation, Ang2 generation, degradation of Ang1, which lead to the pre-administration steady state. So it is rather difficult to evaluate the condition of substrate excess for the second, since the new generation of Ang1 is fast. In addition the reference concentrations *C*_0_ are artificial constructs in a product with *k*_cat_, where both and *K*_m_ are fitted parameters. Thus Michaelis–Menten equations were used, although the *C*_0_ is much higher than the Ang1 concentration for the steady state, as it was used for these enzymes to catalyze this reaction in the described way (Ehlers and Kirsch, 1988; Guillaud and Hannaert, 2010). Although we find an excellent correlation of plasma levels and a good correlation of parameters to the articles cited before, it has not been proven in this article, that the optimized parameter values are unique to reach the reference data, since not for all parameters measured values are available. The Ena model was validated independently from the parameterization of the RAAS and can thus also serve as a validation for the RAAS. Following the development of the Ena model based on literature data, subsequently only population dependent parameters were changed (listed in Table 3) to represent physiological differences in the studies. After coupling to the RAAS model the overall model was able to represent detailed information of all main components and predict circulating plasma concentrations of all included hormones by one coupled PBPK/PD model. The excellent representation of the hormone levels at steady state and during inhibition, implicate a good representation of the underlying physiological processes in the RAAS.

The detailed physiological representation of relevant processes spanning from wb distribution to molecular interactions allows for, e.g., a mechanistic implementation of independent knowledge on drug-target interactions, and thereby an integration and translation of available knowledge.

Thus the model constitutes a first major step to establish a PBPK-PD-model for drugs acting on blood pressure and heart rate. Generally blood pressure and heart rate represent the state of the art endpoint in clinical studies. Currently blood pressure is not yet included in the model. The inclusion of inhibitors from other classes of MoA’s can be used to explore pharmacodynamic effects in the same model framework. Further extensions of this model and an inclusion of the dynamic behavior of the blood pressure in response to changes in RAAS would allow a full mechanistic representation of the PBPK-PD relationship in cardiovascular diseases. Such a model could thus rationalize treatment decisions in the area of cardiovascular diseases. By inclusion of a full process description from administration of a drug to its effect, i.e., the lowering of blood pressure, such a model allows to support cardiovascular drug research from target identification and validation to dosing regimen decision.

## Conflict of Interest Statement

Karina Claassen is funded by and Thomas Eissing, Michael Block, and Stefan Willmann are employes of Bayer Technology Services GmbH, the company that owns and commercializes the software platform used for the simulations described in the manuscript (PK-Sim^®^ and MoBi^®^), as well as parent company stock owners.
